# Gap Junctions and Connexins in Cancer Formation, Progression, and Therapy

**DOI:** 10.3390/cancers12113307

**Published:** 2020-11-09

**Authors:** Randall Ruch

**Affiliations:** Department of Cancer Biology, University of Toledo Health Science Center, Toledo, OH 43614, USA; randall.ruch@utoledo.edu

The development of many forms of intercellular communication was essential for the evolution of multicellular organisms. The organization of cells into tissues and organs and the coordination and homeostatic mechanisms that regulate their cell number, size, and function are highly dependent upon local and distant forms of communication between cells. This cellular crosstalk occurs via direct contact between neighboring cells or the extracellular matrix using various types of junctions, attachments, and cellular protrusions (e.g., gap, adherens, and tight junctions, filopodia and nanotubes, and neurological synapses). Cells also communicate in non-contact-dependent ways by responding to molecules that are secreted by other cells locally or distantly (e.g., growth factors, hormones, inflammatory mediators, and other cytokines) via receptor and non-receptor-mediated processes. Finally, cells secrete exosomes and other microvesicles that carry chemical information to nearby and distant cells. All of these forms of intercellular communication are necessary for the normal physiological activities and homeostatic mechanisms of healthy multicellular organisms and when defective result in the loss of homeostasis, disease, and ultimately death.

This Special Issue is focused on the role of gap junctional intercellular communication (GJIC) and gap junction proteins (connexins) in the biology, causation, and therapy of human cancer. Gap junctions are a unique form of cellular junction in that they primarily serve as aqueous pores or channels that connect directly the interiors of adjacent cells. The diameter of these channels is approximately 1.5–2.0 nm. This size enables molecules and ions of approximately 1.2 kDa and smaller to freely diffuse between two gap junction forming cells, but excludes larger molecules such as proteins and lipids. This facilitates intracellular homeostasis of these molecules and also coordinates their activities through sharing of second messengers and other mediators. Several decades ago, the pioneering work of Loewenstein and Kanno [1] and subsequently many other labs demonstrated that cancer cells frequently exhibited few or abnormal gap junctions and had diminished GJIC. These studies led to the widely held view that defective GJIC contributes to the loss of homeostasis and the “rebellious” characteristics of malignant cells: unregulated growth, loss of differentiated characteristics, invasion, and metastasis [2]. Subsequent investigations, however, have shown the story is more complex. Gap junctions may help cancer cells invade, remain dormant at distant sites of metastasis, increase nutrient supply and waste removal within the tumor, and interact with immune cells to escape detection. Connexins also are more than pore-forming proteins, but can participate as anchors for signaling cascades, act as transcription factors, and may be released in microvesicle membranes. The investigations and reviews in this Special Issue cover many of these and other facets of gap junctions and connexins in cancer.

Kazan et al. [3] demonstrated that connexin43 (Cx43) overexpression in human breast cancer MDA-MB-231 cells suppressed tumor formation and metastasis that were correlated with enhanced expression of E-cadherin and zonula occludens 1 and sequestration of beta-catenin at the cell membrane, whereas Cx43 knockdown resulted in elevated N-cadherin and greater invasion. Examination of triple negative breast tumors showed Cx43 was also down-regulated. Similarly, Fostok et al. [4] utilized non-tumorigenic human breast epithelial cells cultured in 2-D and 3-D to show that knockdown of Cx43 led to mislocalization of beta-catenin and Scrib, loss of cell polarity, increased motility and invasion, and activation of ERK1/2 and Rho GTPase signaling mediated through non-canonical Wnt signaling. 

Regarding Cx43 in cancer cell invasion, Piwowarczyk et al. [5] reported that the cholesterol-lowering drug, fenofibrate, impaired the diapedesis of human lung A549 cancer cells across an endothelial continuum. The drug impaired EGF/ERK1/2 and RhoA/Rac1 signaling and disruption of endothelial barrier functions that were induced in the endothelial cells by A549 cells. This suggested the drug might be useful in lung cancer therapy.

Similarly, neoangiogenesis is critical for the growth of a tumor, but the new vessels are often poorly formed and easily penetrated by invading cancer cells. Okamoto et al. [6] reviewed how endothelial cell gap junctions and connexins might affect tube formation, cellular morphology, stiffness and adhesion, and other aspects important in angiogenesis.

Activation of the Src oncogene has long been known to impair GJIC, but Geletu et al. [7] showed that the oncogene has a dual role upon GJIC. They reported that the oncogene when active at high levels suppressed GJIC through the Ras pathway, but enhanced GJIC through the PI3k or Stat3 pathways when active at low levels. Since these latter two pathways are involved in cell survival, their inhibition and subsequent loss of GJIC would confine apoptosis to single cells. 

The phosphorylation status of connexins may also be involved in cell survival. Jacobsen et al. [8] reported that differential serine phosphorylation sites in the cytoplasmic carboxyl-terminus domain of connexin37 (Cx37) impacted GJIC and controlled switching between cell growth phenotypes (cell death, arrest, and proliferation).

Gap junctions may also support tumor metabolism and cell survival through the exchange of nutrients and waste products with neighboring cells. Swietach and Monterisi [9] reviewed this concept and used computational models to show how cancer cells could benefit from forming gap junctions by enabling the cells to exchange bicarbonate for lactate that would support their anaerobic metabolism that is often seen in poorly perfused tumors.

Gemel et al. [10] demonstrated the presence of Cx43 in exosomes and considered how this might be involved in fusion of the exosome with its target cell, or signaling and transmission of exosomal contents. Shimaoka et al. [11] reviewed other recent findings of connexins and integrins in exosomes and how the proteins might interact to impact fusion and signaling with target cells.

Cancer stem cells (CSCs) are a subpopulation of cells within a tumor that are highly tumorigenic, self-renewing, and resistant to therapy; eradication of these cells may be essential to curing a patient with cancer. The role of gap junctions in CSCs has only recently begun to be investigated. Beckmann et al. [12] reviewed this recent literature. Trosko [13] further expanded upon this CSC/GJIC relationship by suggesting two types of CSCs may occur (those that express connexins, but still exhibit defective GJIC with normal cells and those that exhibit no connexin expression). He also posited these two types of CSCs may be derived from normal adult stem cells that similarly do not express connexins or that express them, but GJIC is absent due to oncogene activation. Ruch [14] showed in highly malignant human lung carcinoma cells that ectopically expressed Cx43 suppressed several CSC-phenotypic characteristics (invasion, migration, colony and tumorsphere formation, expression of pluripotency transcription factors, ALDH expression, and increased sensitivity to the anti-cancer agent cisplatin). These results suggest the upregulation of Cx43 may be a viable approach to target lung CSCs.

The assembly and function of gap junctions and localization of connexins are altered by many types of carcinogens and therapeutic agents [15]. Using confocal laser microscopy and single molecule localization microscopy, Pilarczyk [16] reported how neuregulin, trastuzumab, and 4 Gy irradiation impacted Cx43 localization in human fibroblasts, mammary endothelial cells, and breast cancer cells.

The bystander effect in cancer therapy involves the death of cells that are not attacked by a therapeutic agent or radiation, but that are adjacent to targeted cells. This involves the movement of poorly characterized, “death-inducing” molecules from the latter cell to the former through gap junctions or the extracellular space. Arora et al. [17] showed that bystander killing occurred after cisplatin treatment of human lung cancer cells and that this was dependent upon GJIC and “death signals” that induced DNA damage (double strand breaks) in the bystander cells.

Romo et al. [18] investigated the effects of non-genotoxic, low molecular weight polyaromatic hydrocarbons that are found in cigarette smoke on GJIC in lung epithelial cells cocultured with alveolar macrophages. These agents may contribute to lung carcinogenesis by inhibiting lung epithelial GJIC. The agents caused the loss of GJIC and this was prevented by anti-inflammatory parthenolide. This suggests inflammation also contributes to impaired GJIC and lung carcinogenesis.

Several papers considered the roles of gap junctions in various types of human cancer. Tschernig [19] considered gap junctions in bladder and other urinary tract cancers. Asencio-Barria et al. [20] considered the importance of not just gap junctions, but also exosomes and tunneling nanotubes in prostate cancer. Kiszner et al. [21] characterized the expression of several connexins and their localization in a progression of cells from primary melanocytes to common and dysplastic nevi to metastatic melanoma. They reported changes in Cx26, Cx30, Cx32, and Cx43 that were associated with tumor progression and heterogeneity within tumors. Aasen et al. [22] used various in silico tools and TCGA data to compare connexin expression in lung cancer. They found connexin expression was correlated with histological subtype of the cancer and was either up- or downregulated depending upon the connexin considered. They also found Cx43 nuclear localization correlated with poor survival.

Lastly, Le Vasseur et al. [23] looked at another type of channel forming protein, pannexin 2, and its localization within cells. Pannexin 2 normally localizes to the cell membrane and forms hemichannels that open to the extracellular space. The protein also has tumor suppressing activity. Using several techniques, the authors showed the protein could be found at sites of ER-mitochondria contact and further made the cells more sensitive to apoptosis.
